# Integrated DC resistivity and seismic refraction imaging for bedrock discontinuity mapping in New Minia city, Egypt

**DOI:** 10.1038/s41598-026-61212-3

**Published:** 2026-07-20

**Authors:** Kh. S. Gemail, M. Attwa, S. Shebl, Shokry A. Soliman, A. Azab, Mohamed H. Farag

**Affiliations:** 1 Environmental Geophysics Lab (ZEGL), Faculty of Science, Geology Department, Zagazig University, Zagazig, 44519 Egypt; 2 Faculty of Science, Geology Department, Zagazig University, Zagazig, Egypt; 3 Division of Geological Applications and Mineral Resources, National Authority for Remote Sensing and Space Sciences, Cairo, Egypt; 4 Geophysics Laboratory, Exploration Department, Egyptian Petroleum Research Institute, Nasr City, Cairo, Egypt; 5 EPRI Core Analysis Center, Egyptian Petroleum Research Institute (EPRI), Nasr City, Cairo, Egypt

**Keywords:** DC resistivity, ERT, P-wave, Bedrock discontinuities, Geotechnical site characterization, New Minia city, Engineering, Environmental sciences, Natural hazards, Solid Earth sciences

## Abstract

This study addresses geotechnical challenges in fractured limestone environments by integrating electrical resistivity tomography (ERT), seismic refraction, and direct current (DC) resistivity soundings with borehole data in New Minia city, Upper Egypt. The objective was to delineate shallow subsurface heterogeneity and structural discontinuities and to establish correlations between geophysical and geotechnical parameters. A genetic algorithm (GA) optimization was applied to DC resistivity inversion, reducing non-uniqueness and improving model convergence. Seventeen boreholes (10 m depth) provided constraints for calibration and validation. Results reveal three principal subsurface sequences: (1) sandy–silty soil with clay intercalations, (2) heterogeneous intermediate deposits, and (3) fractured limestone bedrock. Major structural features, including north–south trending faults, were clearly identified. The integrated approach enhances subsurface characterization, reduces interpretational uncertainty, and provides a robust framework for hazard assessment and urban planning in complex carbonate terrains.

## Introduction

A geological discontinuity is a sharp boundary between two different geologic units that is recognized based on a lithological, textural, structural, or sequential variation between units, such as a stratigraphic contact (i.e., soil and rock), or a structural discontinuity such as a fault or fracture zones. The stability degree of limestone bedrock is variable according to lithological discontinuity and geological structure, as well as the occurrence of clayey bodies ^1^ and ^2^. In such a case, the geotechnical site characterization needs a full imaging representation of near-surface stratigraphy (including short-distance variability) to evaluate the geotechnical properties of soil and bedrock. Such features are often sub-vertical and therefore difficult to locate by drilling, even when the boreholes are closely spaced ^3^.

**Fig. 1:**
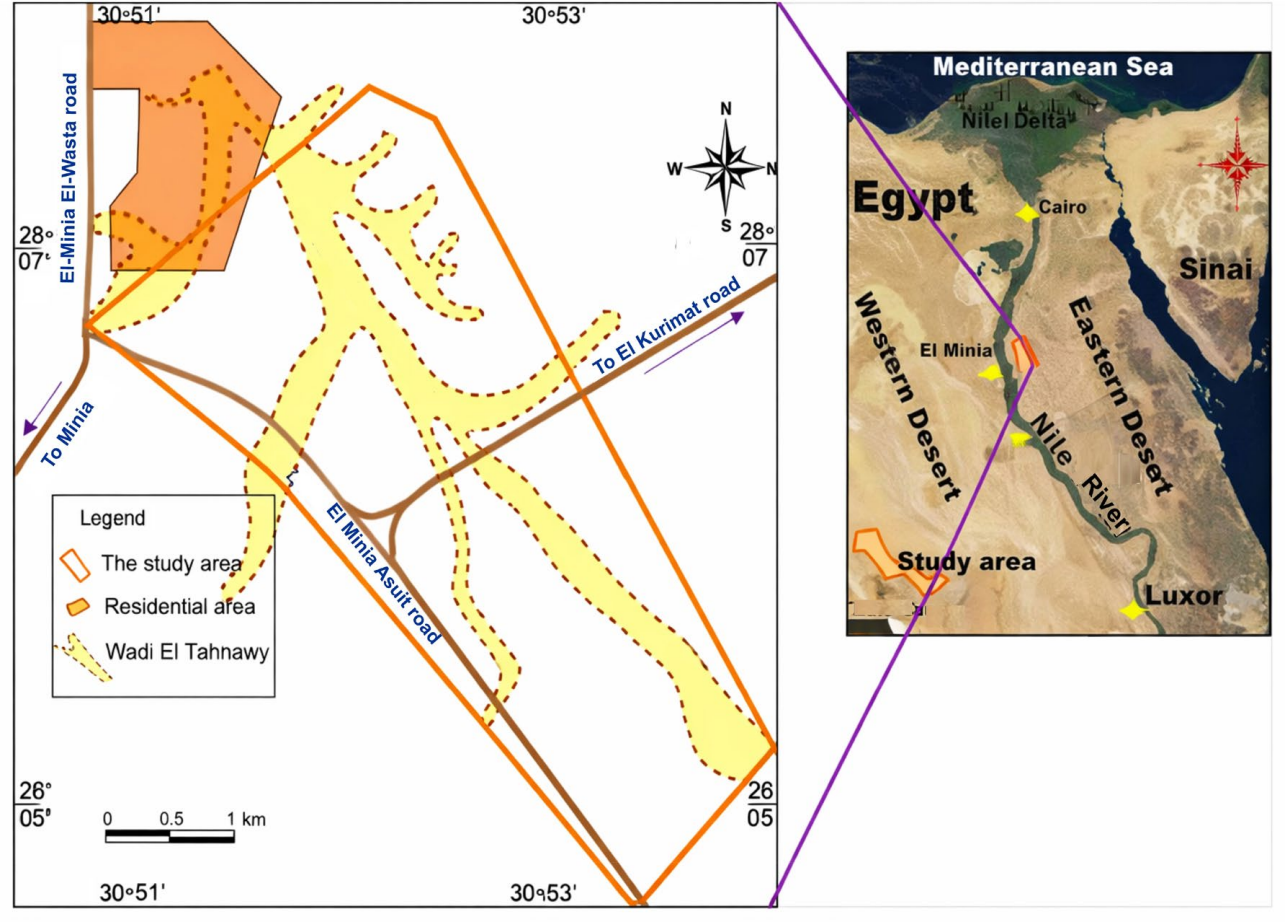
Location map of the study area and drainage networks in New Minia.

**Fig. 2:**
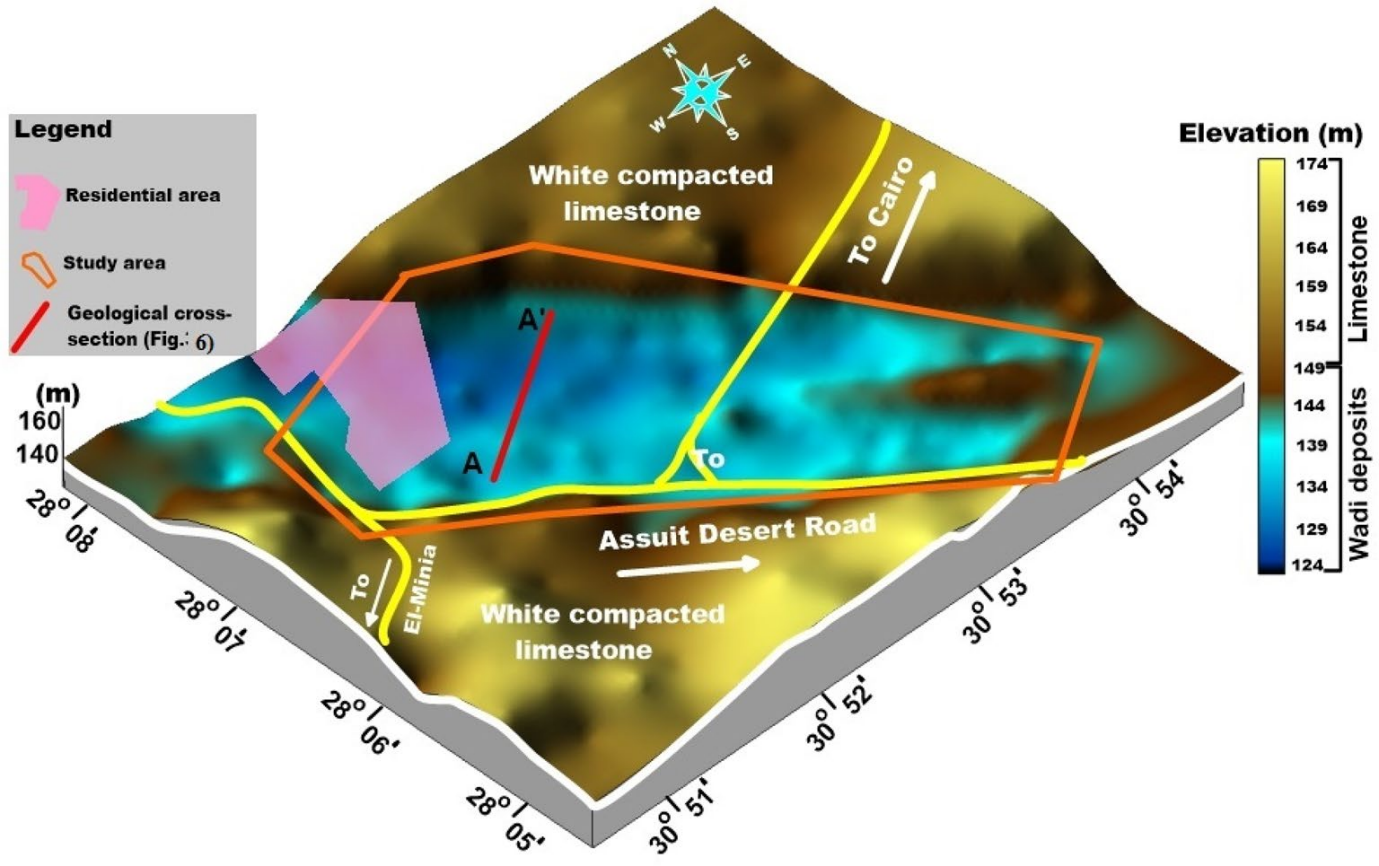
3D topographic block model of the New Minia area, highlighting elevation ranges (124–174 m), drainage pathways, and limestone exposures.

In the present work, New Minia city is considered a case study for mapping the bedrock discontinuities, which are common in the limestone bedrock. New Minia city, situated on the eastern side of the Nile River, about 250 km south of Cairo, covers an area of 11 km^2^ (Fig. 1). Urban expansion in New Minia city needs more site investigations to determine the suitability of the foundation bedrock in the extended sectors. Karst and caverns situations and structural elements (i.e., fault, joint, and fractures) in the local heterogeneous limestone bedrock limit its suitability for foundation purposes ^4^. The limestone bedrock in the area is highly cavernous and dissected by a large number of minor faults and joints, which cause instability of the foundation bedrock ^5^. Regionally, major NW–SE and NE-SW trending faults control the main drainage lines and escarpment boundaries in the area (Fig. 1). The rock discontinuity in the area is structurally controlled, mainly by a NW–SE fault trend and partially by the N–S and NE trends ^6^. From a topographic perspective, the area lies on a shallow surface drainage system (Fig. 2). The topographical hazard is increased by fracturing, and it should be considered if the region is subjected to heavy rainfall for a long period or urbanization activities.

Geophysical techniques, including DC resistivity and seismic refraction imaging, when integrated with geotechnical investigations, offer a detailed understanding of near-surface conditions, and may even reveal several types of site characterizations that typical geotechnical methods, like drills or field investigation, might fail to detect ^7–10^.

The 2D electrical resistivity tomography is a valuable technique for interpreting the subsurface geological structures, while the seismic method is suitable for mapping bedrock depths and fracture zones, but fails to map low velocity layers at great depth or thin beds between two high velocity layers, as does the 2D resistivity method. Thus, the combined use of both techniques will give more accurate results for delineating buried geological discontinuities and structural features for engineering purposes.

2D resistivity imaging is one of the geophysical survey techniques often applied in an early stage of site investigations to get a first-order idea of the shallow lithologic discontinuity ^1,11–15^. Thus, the objective of the present work is to map and understand the problematic conditions of the subsurface bedrock and confirm its features using a combination of borehole exploration, resistivity, and seismic techniques, and to investigate the subsurface materials and structures, which are likely to have considerable engineering implications at the New Minia city. The combined use of geoelectrical resistivity and seismic refraction techniques for site investigations is very effective because each method has a distinct response to a physical property with varying resolution ^16–18^.

Geophysical techniques provide continuous subsurface coverage, while boreholes offer point-specific calibration. In this study, 17 boreholes were systematically integrated with DC resistivity soundings and seismic refraction profiles, ensuring that inversion ambiguities were minimized through direct lithological control.

Recent advances in integrated geophysical site characterization have demonstrated the value of combining ERT with seismic methods for karst detection ^19–23^. Innovations such as genetic algorithm optimization of resistivity inversion ^24^ highlight the methodological contribution of the present study, which applies GA-based inversion in a carbonate terrain with complex discontinuities.

## Geological and geotechnical background

**Fig. 3:**
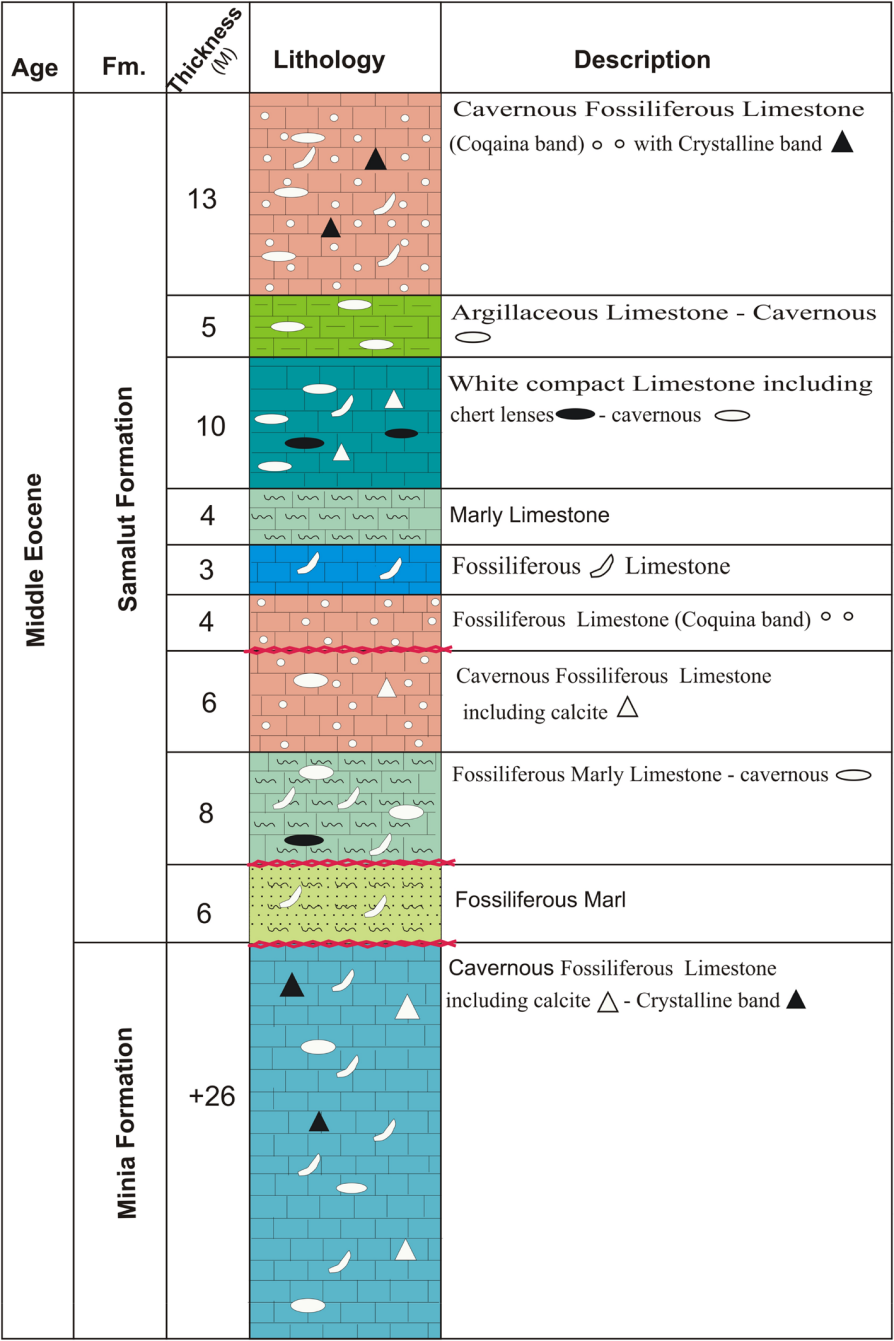
Lithologic sequence of the New Minia city shows the main rock units (modified after ^6^).

Understanding of the geological and geotechnical properties of soils and rocks in the near-surface zone and identification of different geological hazards play an essential role in planning and constructing infrastructure in new urban areas in order to avoid any damage to the constructed projects (^25^ and ^26^). In the New Minia city, Middle Eocene carbonate rocks and Quaternary Aeolian with fine deposits cover the area (Fig. 3). These rocks are mainly formed of cavernous and inhomogeneous fossiliferous limestone with chalk intercalated and thinly interbedded clayey, sandy, and cherty limestone layers ^27^. The limestone bedrocks are covered, in some locations, by conglomerates, fine Aeolian deposit sand talus. The bedrock is highly cavernous and separated by a large number of minor faults and joints, which cause instability of the foundation bedrock.

**Fig. 4:**
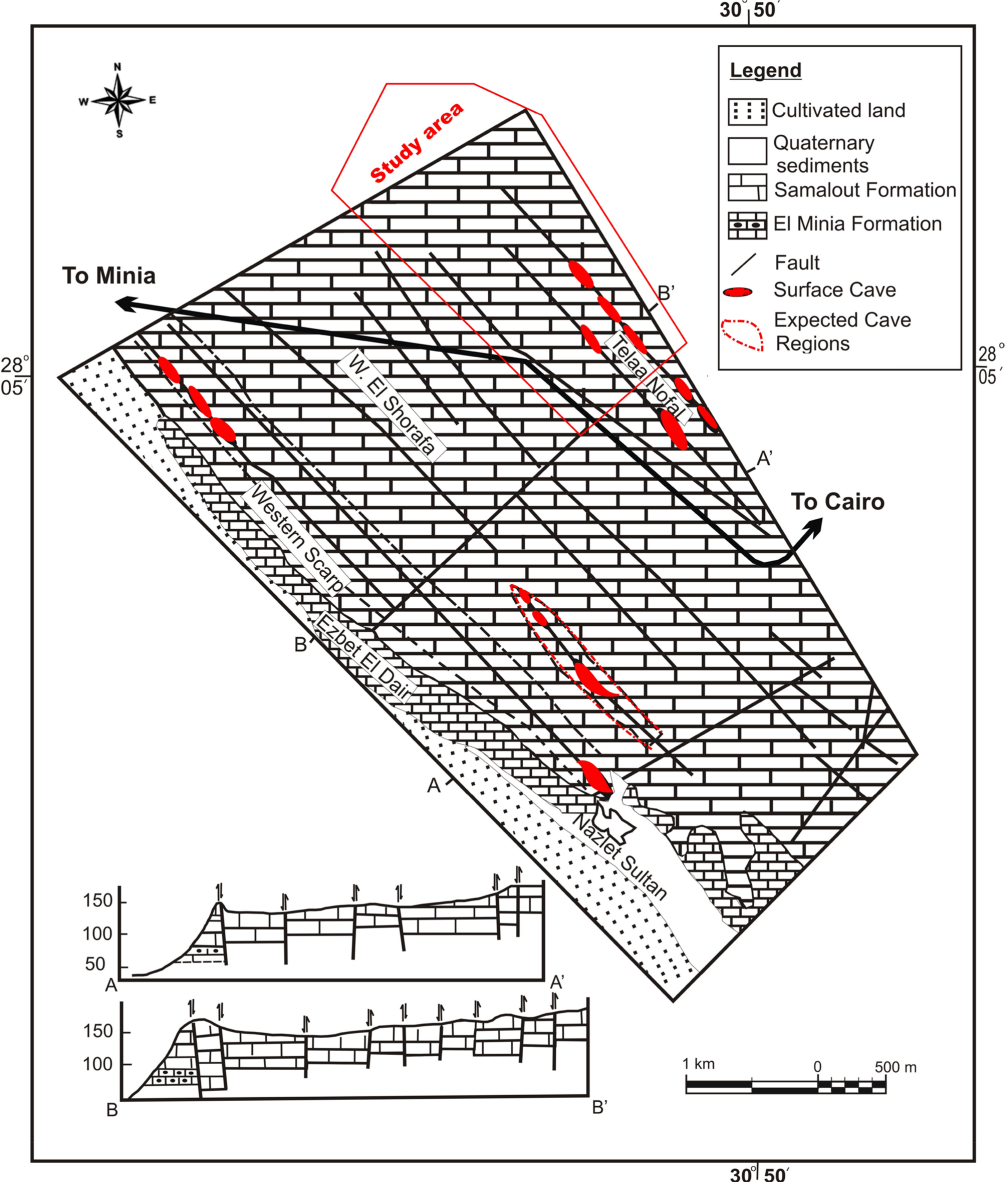
Detailed structural map and two geological sections show subsurface layer distribution in the New Minia city (after ^6^).

The detailed geological studies (i.e., ^5,6,28^, and ^4^) revealed the presence of deep caves in the building site of the city (Fig. 4). These caves may be formed due to rainwater or groundwater passing through NW–SE faults and fractures and the dissolution of soluble materials in the limestone, as shown in the structural map of New Minia city (Fig. 4).

In the geological map of the area (Fig. 4), the carbonate rocks are divided into two formations from top to bottom: Samalut Formation and Minia Formation ^29^. The Minia Formation is the deepest and formed of intercalated chalk and cavernous limestone rocks, which are cross-bedded and contain flints at its bottom ^6^. The topmost bedrock consists of Samalut dense and karstic limestone with numerous sinkholes and caves, which are sometimes linked together (Fig. 4). This limestone bedrock is formed of a Pale yellow to white limestone and chalk containing some clay, marl intercalations, and coquina beds. At the base of the Samalut Formation, many vugs, voids, and caves are distributed almost oriented NW–SE, aligned with the main faults. This may be due to the influence of surface and/or groundwater flow along fractures where permeability increases ^30^.

**Fig. 5:**
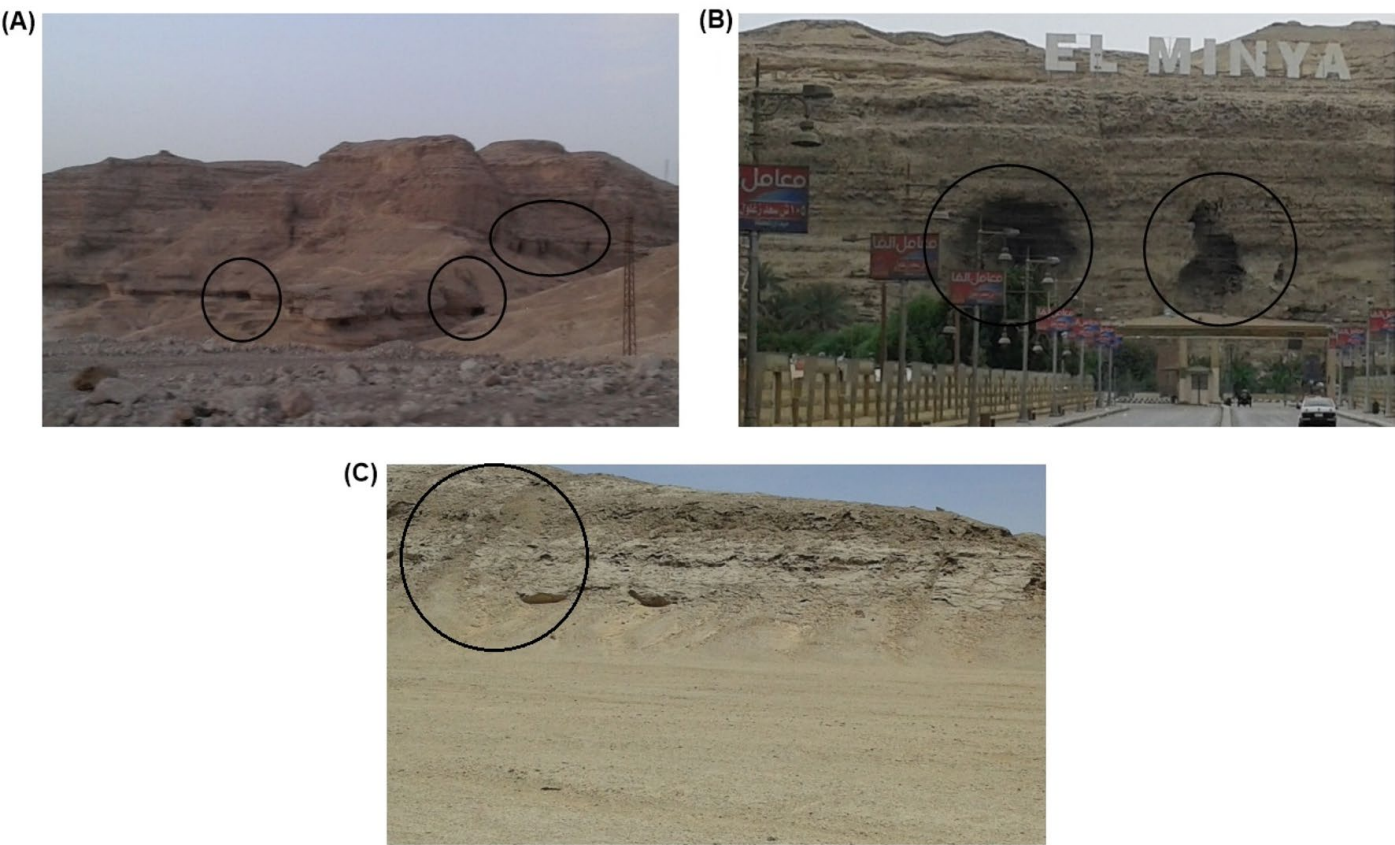
Photographs of the limestone exposures in the area show a) karstic limestone with numerous vugs, holes and caves b) large caves in cavernous fossiliferous limestone c) Highly fractured limestone in the surveyed area.

Structural setting, as illustrated in Fig. 4, revealed that the New Minia site is subjected to sets of normal faults and fractures trending in NW-SW and NE-SW direction, where the NW faults are dominant ^6^. Most of the caves and sinkholes in Samalut Limestone are distributed along major faults with different sizes (Fig. 4). The lengths and extensions of these caves range from few meters to more than one kilometer (Fig. 5). These fractures increase along the major faults where caves, frequency and size also increase and the combination of fractures and caves may increase the hazardous conditions in the area foundations. Additionally, fractures and joints may act as channels for surface and groundwater, which can dissolve the limestone, affecting the stability of the bedrock due to the relative decrease in shearing resistance.

**Fig. 6:**
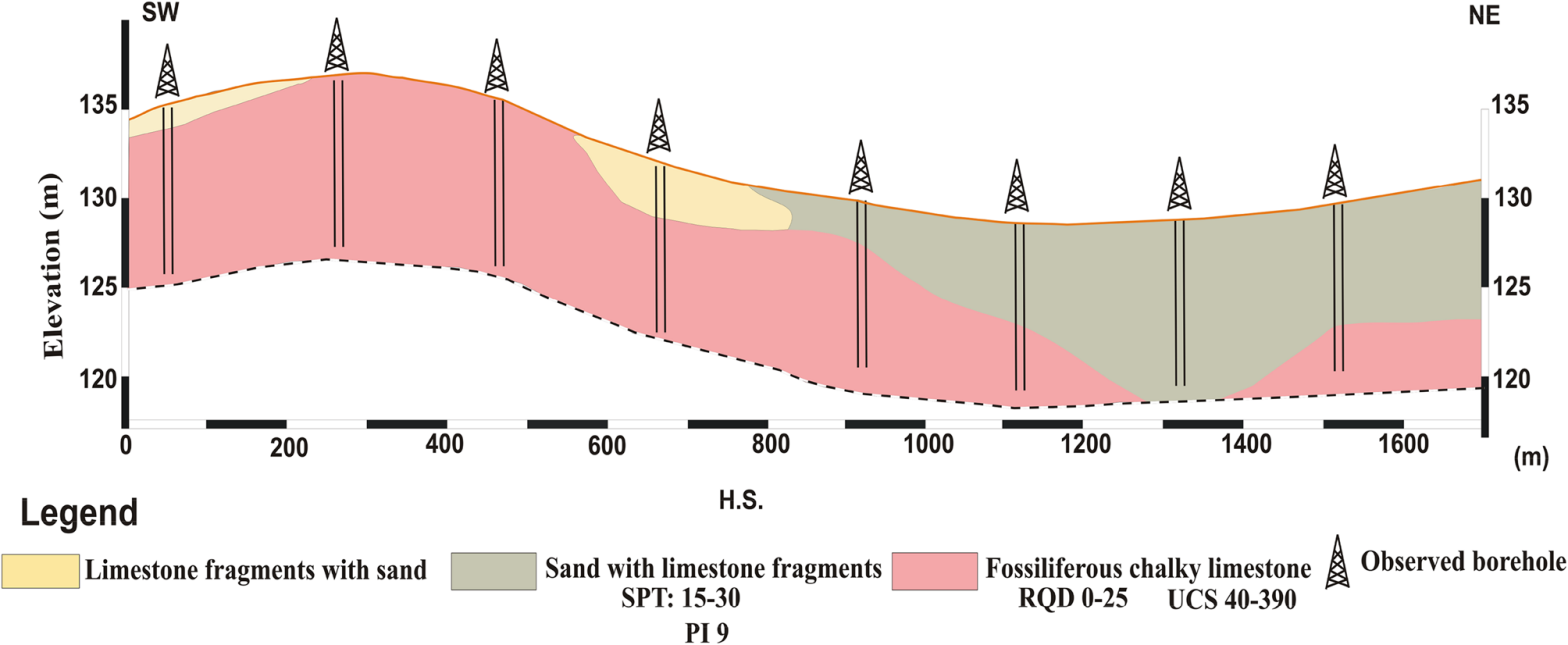
Geological cross section in the northern part of surveyed area shows the discontinuities in the limestone bedrock based on shallow drilled boreholes (for location see Figs. 2 and 7).

According to their geotechnical and engineering properties, ^31^ classified both the limestone units per ^32^ engineering classification as class K III, where the local limestone layers have many caves and dropout sinkholes, in addition to large dissolution patterns along the major fault lines with small collapse and widespread fractures. To illustrate the lateral variations in the topmost parts of the area, a geological section was constructed using ten-meter boreholes drilled by Arab Contractors ^33^ as shown in Fig. 6. Accordingly, the area could be discriminated into four lithologic units with different geotechnical conditions. This lithology unites, and their geotechnical parameters are represented in the legend of Fig. 6. The fossiliferous cavernous limestone of the Samalut Formation is considered the main foundation bedrock with different Rock Quality Designation (RQD) index. Considering the fractures and sinkholes in this zone, knowledge of the discontinuity and trend of fractures at shallow depths is important to estimate the appropriate and safest depth to place the foundations. In the lowland, the limestone bedrock is covered by silt, sand, and limestone fragments with a thickness ranging between 2 and 10 m (Fig. 6). In places, there are clay and sandy clay interlayers over the limestone bedrock with a maximum thickness of 5 m at 2 m. From a geotechnical and geological point of view, the stability degree of limestone bedrock in the area is variable according to lithological characteristics and structure, as well as the occurrence of clayey interlayers. In such a case, there is a great need to understand the distribution of such rock discontinuity, in view of geotechnical properties as foundation bedrock.

## Geophysical data acquisition

**Fig. 7:**
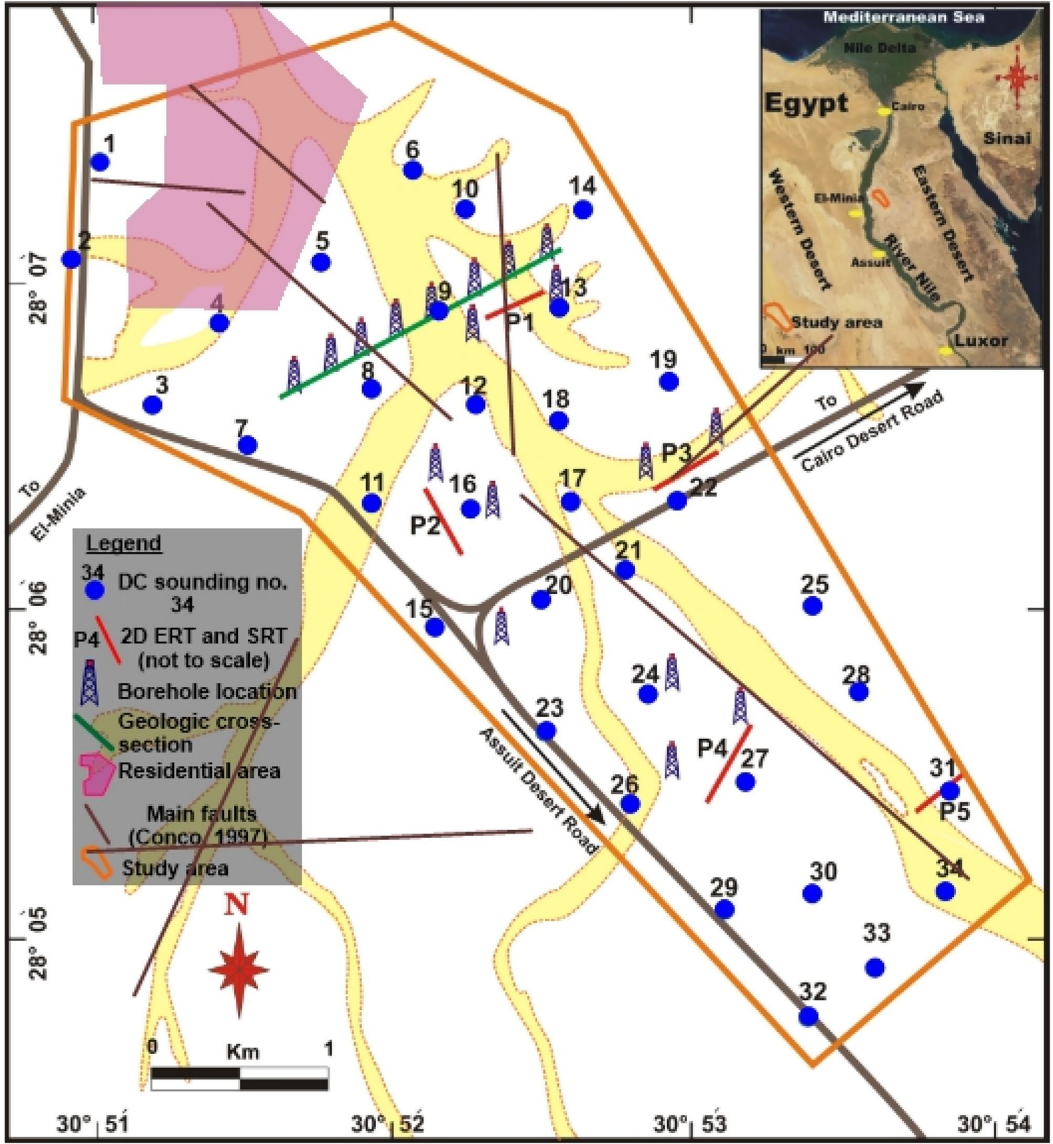
location map of geophysical measurements and drilled bore holes in the study area.

The geophysical survey in the form of DC resistivity and seismic imaging was conducted mainly to detect rock discontinuities and provide an analysis of the data in relation to the foundation rock materials and parameters as much as possible. In the present surveys, the DC resistivity measurements were acquired by applying the Schlumberger vertical sounding technique to map the bedrock and soil distributions in the area. The Schlumberger electrode array was selected with 600 m maximum current spacing (AB), which is characterized by great penetration depth and its sensitivity for heterogeneities in the surface layers ^33^. According to the available geological and geotechnical information from the drilled boreholes by Arab Contractors ^33^, the applied electrode spacing is long enough to cover the silt and sand overburden and reach the limestone bedrock in the surveyed area. The apparent resistivities were collected at 34 sounding points as the first stage of the field measurements using SAS 300C terrameter with repeatability of 4 cycles to control the measurement accuracy. These sounding points were measured with unequal spacing based on the field circumstances and the expected bedrock, as shown in Fig. 7.

According to the interpreted vertical contacts in the limestone bedrock from the prior Schlumberger soundings, five sites were selected for more detailed mapping using seismic refraction profiling and electrical resistivity tomography (ERT). The ERT and refraction seismic profiles were executed due to short-distance variations in the subsurface conditions as indicated from the former 1D sounding survey of the first measurements stage. Resistivity and seismic refraction profiles were correlated with borehole data that were inline and nearest to the survey lines (Fig. 7).

In the ERT survey, Wenner Beta (WB) was selected, which is basically a dipole–dipole (DD) array with a dipole distance equal to the dipole length. Both arrays (WB and DD) are the most sensitive to horizontal changes in the subsurface resistivities, such as in the case of vertical structures like faults, fractured zones, and cavities, which are common in the surveyed area. In comparison, the WB array has a better signal strength at great depths than the DD array, where the thickness of the silt and sands overburden extends to 10 m in some places. Additionally, the limestone bedrock changes to silty clay and argillaceous and cavernous limestone at different depths in the area ^4^. These conditions reflect the reliability of the WB array to detect the vertical contacts and heterogeneity in the limestone bedrock and soil overburden.

The WB profiles were carried out using Siber-48 (48 channels) multi-electrode automatic system to reduce the systematic errors during measurements as much as possible. The apparent resistivities were measured along a 235 m profile with 5a m minimum electrode distance and data level n = 15. These parameters are suitable for imaging the variations in the bedrock to a depth of 30 m. During the field work, the instrument obtained the apparent resistivity and estimated the standard deviation errors (Q %) from repeat cycles. The measurements with a standard deviation error of more than 5% were repeated or rejected.

Along the measured WB profile, seismic refraction profiles were executed to confirm the bedrock boundaries and detect the discontinuities in the area. In addition, 25 seismic lines were distributed to cover the whole area. The shallow seismic refraction data acquisition was conducted using the 24-channel McSeis-SWX. The geophones (24) were arranged along a 115 m profile with 5 m spacing. Five shots were carried out at each line, and each shot was stacked to improve the arrivals at distant geophones, improve data quality, and reduce the impact of traffic noise at each geophone. To generate the P-waves, a 10 kg hammer was used with a metallic plate (20 × 20 cm^2^) to receive the sledgehammer strikes. A total of 4 to 5 stacks were employed at each P-wave shot point.

For geotechnical data, the geotechnical report of Arab Contractors ^33^ was referred including soil and rock samples from 17 boreholes with a maximum depth of 10 m. The geotechnical data were employed for soil and bedrock physical modelling and verifying geophysical data, and include Rock Quality Designation (RQD), Unconfined Compressive Strength (UCS), Moisture Content (WC %), and SPT N-value.

## Geophysical data inversion

The measured Schlumberger soundings were processed and interpreted using the IP2WIN program ^34^. The 1D modeling process in this program uses the linear filtering algorithm, which offers a fast and exact direct solution for a large number of models to cover the most reasonable geologic situations. During the inversion procedures in this program, the resistivity soundings along the profile were treated as a unit representing the subsurface geological structure of the surveyed area as a whole, rather than a set of independent objects. Data quality was ensured through repeated measurements, rejection of outliers, and error analysis. The Wenner Beta array was chosen for its superior depth penetration and signal strength in clay-rich overburden. GA-based inversion further reduced non-uniqueness, improving correlation with borehole data. Additional geological and geotechnical data from boreholes with 10 m depth ^33^ were employed to adjust the obtained models and outline the lithologic boundaries in the area. The obtained models represent multi-layered structures with five layers, as shown in Fig. 6.

**Fig. 8:**
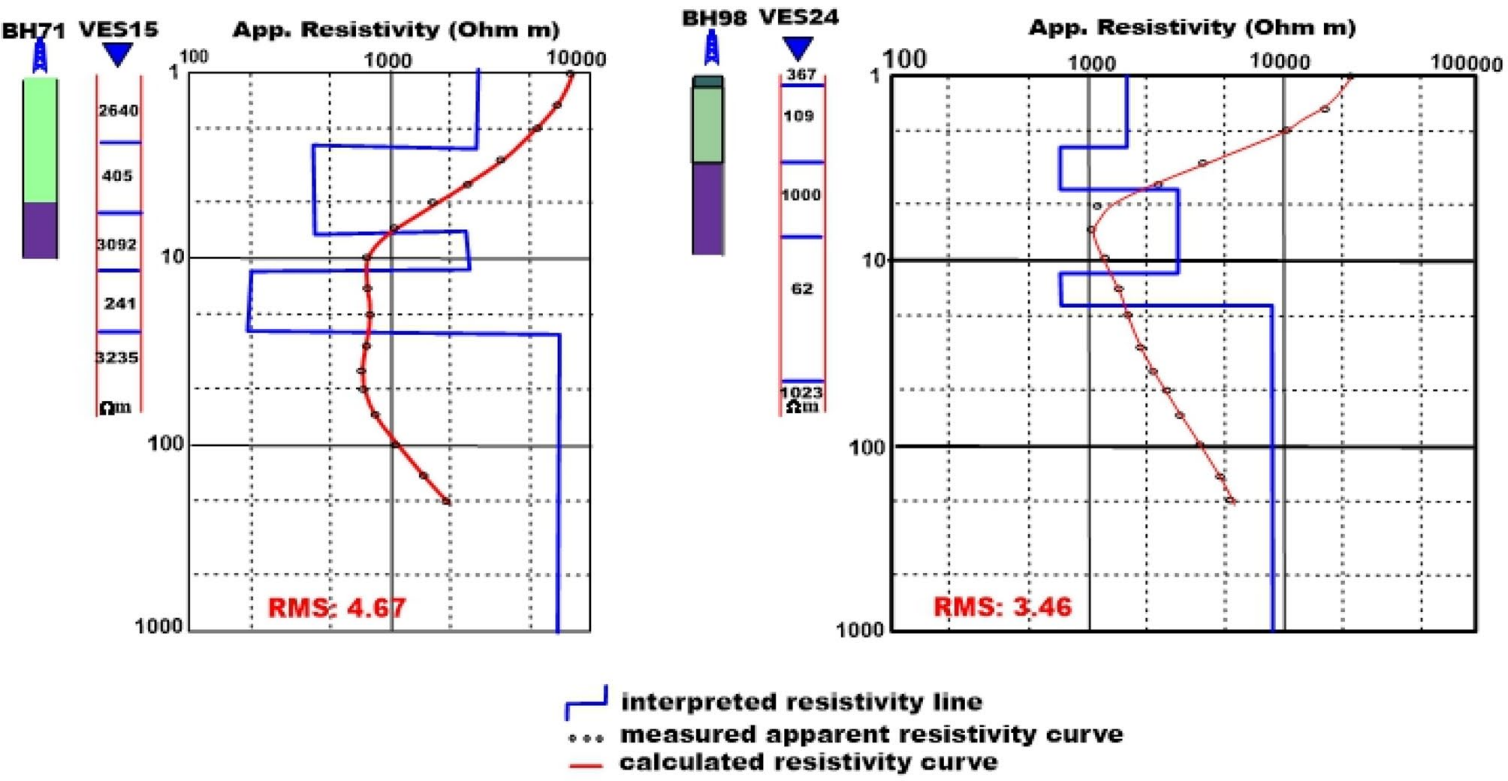
Correlation between interpreted resistivity models of soundings 15 and 24 with boreholes BH71 and BH98, located in the central sector of New Minia (see Fig. 7). Array: Schlumberger, AB/2 up to 200 m.

The principle of equivalence was taken into account during the inversion process, where the limestone as resistive in some places, sandwiched between two layers of lower resistivity (upper silty clay and argillaceous limestone in the lower). Similarly, a conductive clay layer (2–6 m thick) lies in between two resistive layers of dry sand and gravel in the upper and fossiliferous limestone in the lower ^33^. On the other hand, the conditions of the suppression principle are reported in some boreholes where a relatively thin layer of clay or limestone squeezes between two layers of increasing or decreasing resistivity. In such cases, the geological interpretation of 1D inversion is tricky and needs some considerations, such as fixing some model parameters (depth or resistivity of layers) according to the available geological information ^34^, which was obtained from available boreholes (Fig. 8).

**Fig. 9:**
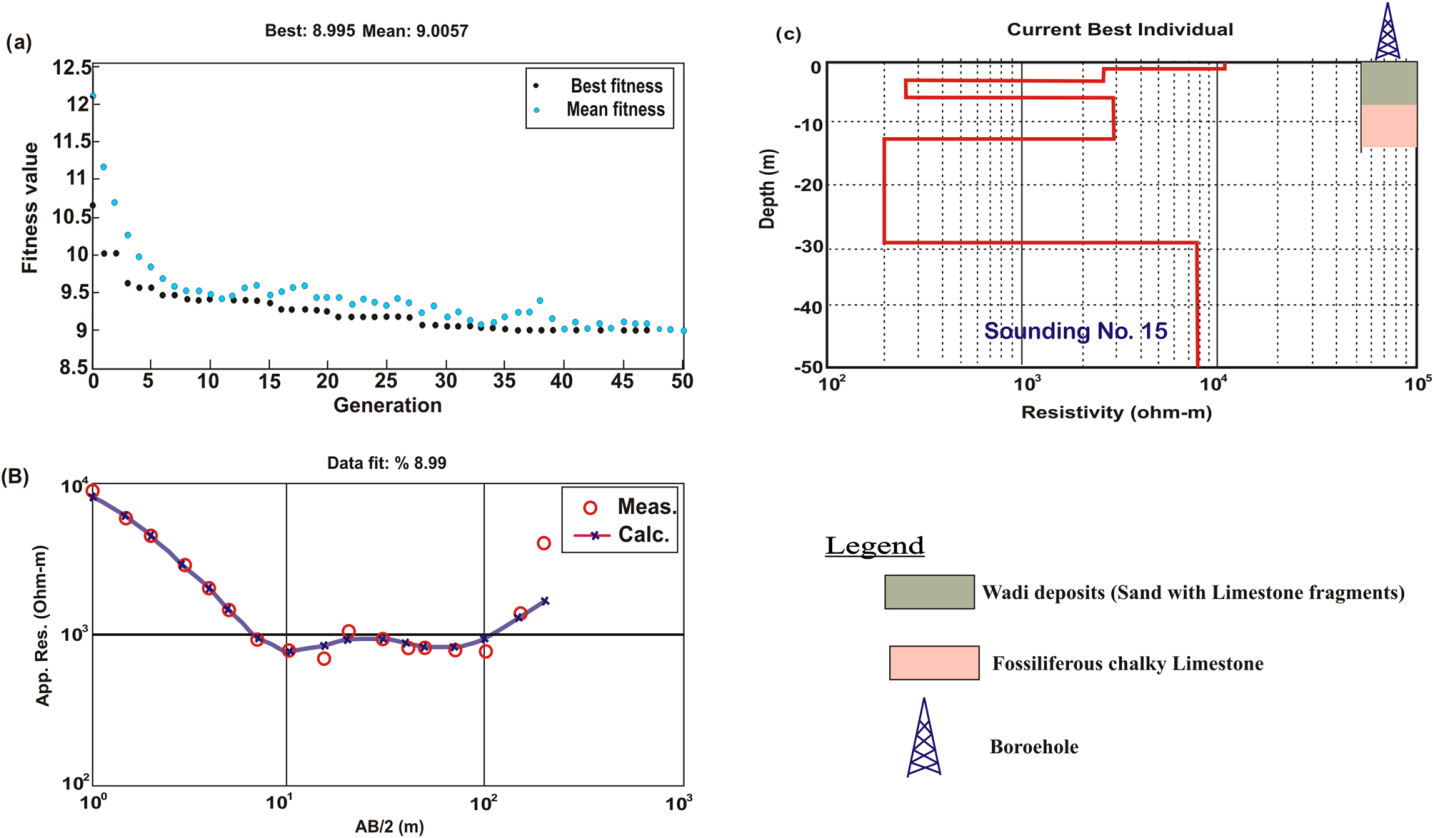
Inversion results applying GA of DC RESISTIVITY sounding point No. 15 (for location, c.f. Figure 7) using GA with borehole data calibration; the population size and generation number were 50.

To reduce the uncertainty inherent in direct current resistivity soundings, particularly the limitations of 1D inversion related to equivalence and suppression effects, the data were pre-processed using a MATLAB in-house code ^35^ to remove spurious values. The apparent resistivity data were then inverted through a sequential application of conventional linear filter inversion ^34^ and a non-conventional genetic algorithm (GA) scheme (^36^ and ^37^). While the conventional approach relies on a single starting model, the GA employs multiple starting models within a broad search space, thereby increasing the likelihood of reaching a global minimum solution ^38–40^. This procedure provided improved model reliability and better correlation with borehole data, as illustrated in Fig. 9, the inversion process using a genetic algorithm (GA) for DC resistivity sounding No. 15 is presented as an example (location shown in Fig. 7). The population size and generation number were set to 50 (Fig. 9a), yielding the best fit between measured and calculated data (Fig. 9b). Furthermore, Fig. 9c demonstrates a clear correlation between the borehole data and the DC resistivity inversion results. The GA inversion results were subsequently used to construct stitched resistivity sections, which were cross-validated with borehole and geological information to ensure consistency. In revising the interpretation, particular care was taken to avoid overstatement and to acknowledge the resolution limits of 1D inversion in areas with pronounced lateral heterogeneity.

Digital subsurface modeling was performed using Rockworks software inverse distance–anisotropic algorithm 14 package ^41^, which provided the computational framework for integrating borehole information with geophysical soundings. The methodological workflow encompassed data preprocessing, inversion of DC resistivity soundings, and preparation of stitched resistivity sections, ensuring compatibility with subsequent visualization steps. Particular care was taken to maintain consistency between borehole records and geoelectrical interpretations, thereby reducing ambiguities inherent in resistivity soundings. This methodological foundation established the basis for constructing the three-dimensional visualization model later presented in the results section.

**Fig. 10:**
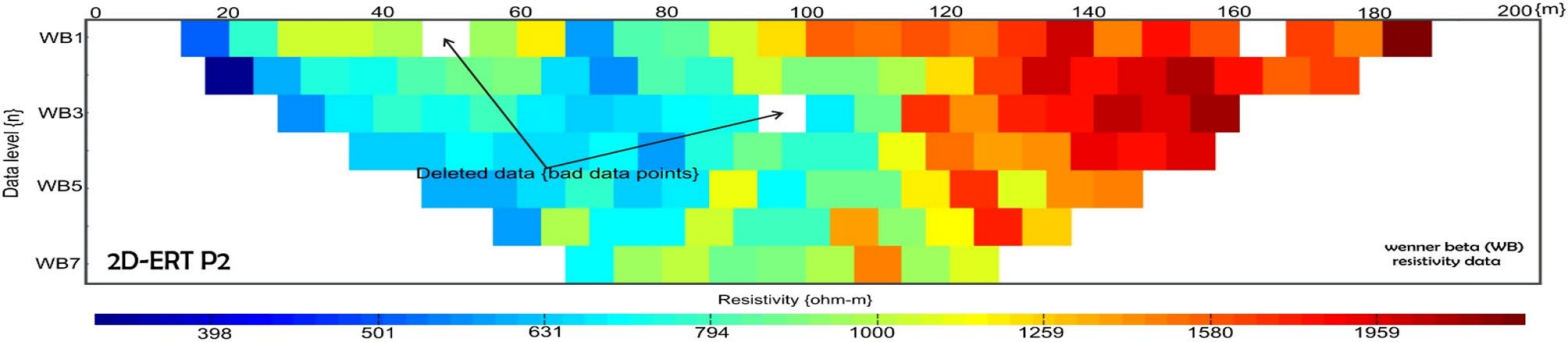
Resistivity pseudo-section of 2D-ERT P2 (for location, c.f. Figure 7). Note that the data levels (n) for Wenner beta (WB) array is 8.

The acquired 2D resistivity data in the study area were processed and inverted using Res2dinv ^42^ and DC2DInvRes ^43^ resistivity inversion software. Both programs are based on numerical modeling techniques using finite-difference (FD) method, which produces an image of the electrical resistivity distribution in the subsurface based on least-squares optimization technique that give reasonably accurate models for a variety of subsurface structures ^44^ and ^45^, Before the inversion routine, the 2D processing included the measured data filtering by rejecting bad quality data-points (Fig. 10), which show abrupt change over the measured points and the stacking error exceeded 1%. Along the measured profiles, the removed points did not exceed 5% of the total number of measurements.

**Fig. 11:**
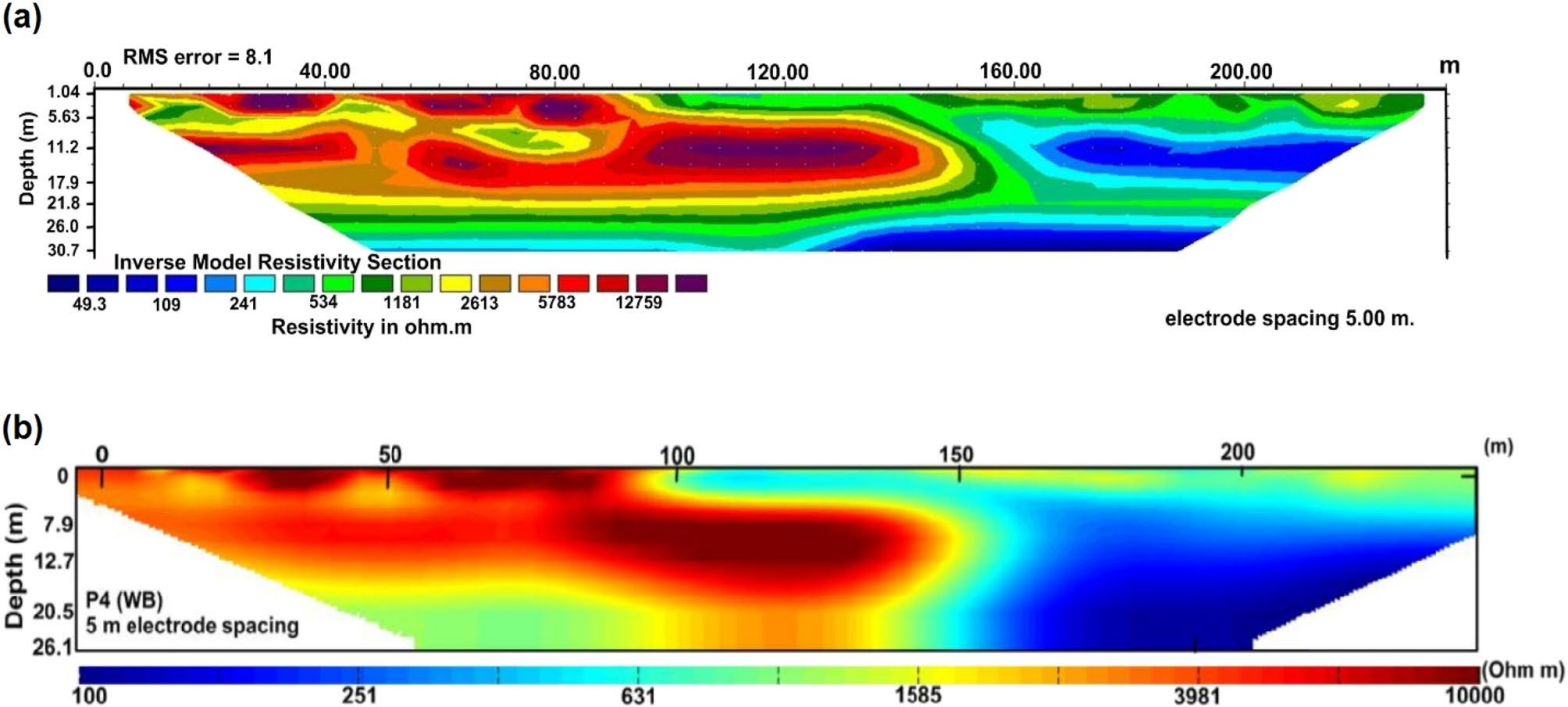
2D inversion model for forward WN array (P4), inverted by (a) Res2dinv ^36^ and (b) DC2DInvRes ^37^ softwares (for profile location, see Fig. 7).

Although there is a general agreement between the inversion results of both Res2dinv and DC2DInvRes programs, the layer boundaries can be easily detected from the DC2DInvRes 2D image than from the Res2dinv inverted sections. Furthermore, the penetration depth may be detected freely depending on the sensitivity analysis using DC2DInvRes compared with Res2dinv. Consequently, a higher resolution 2D image can be noticed at Fig. 11.

**Fig. 12:**
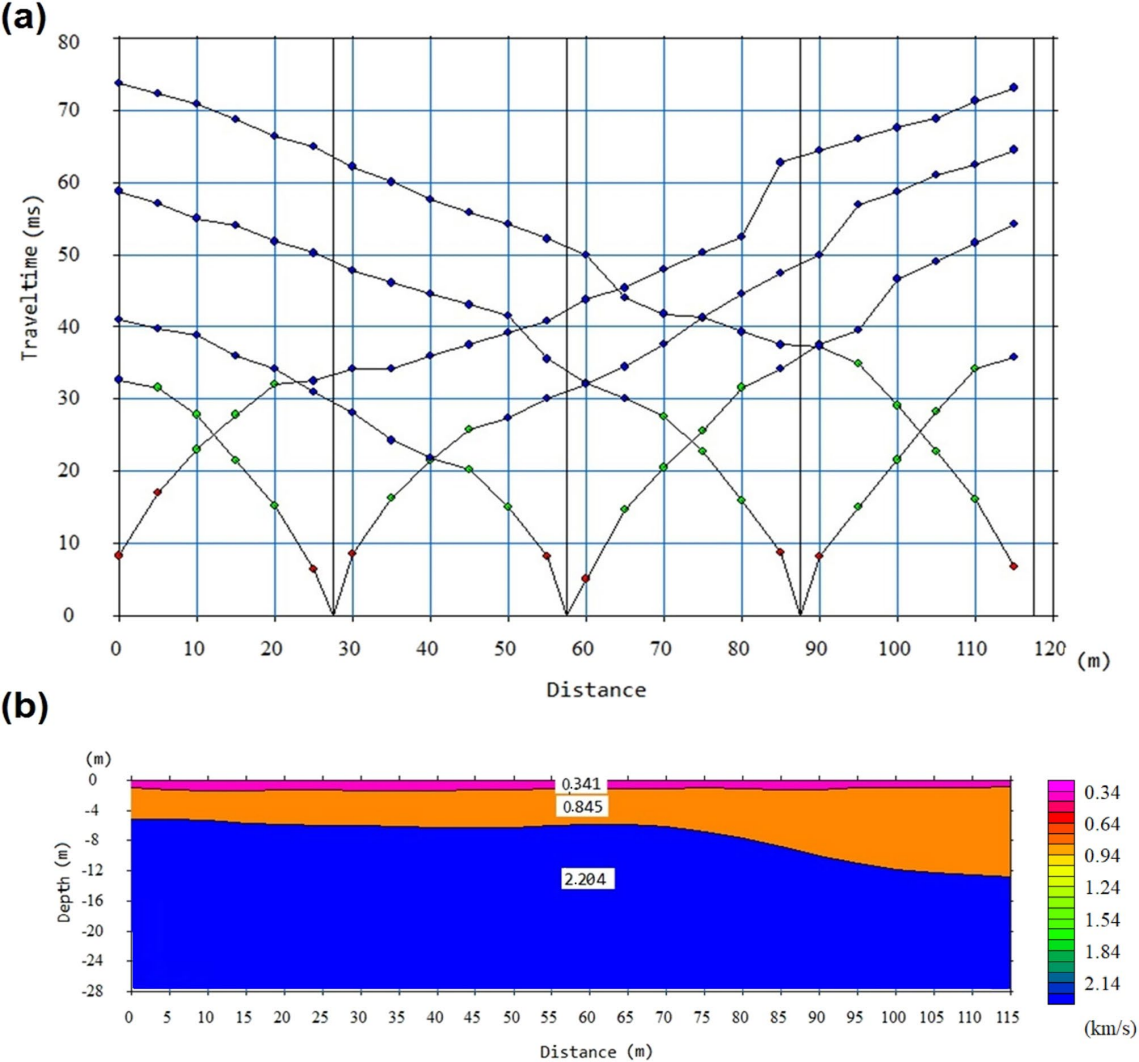
Seismic profile 3, located in the central part of the New Minia survey area. (a) P-wave first-break picking from 24-channel refraction data, acquired with 5 m geophone spacing. (b) Final velocity–depth model to ~ 30 m depth, showing fractured limestone bedrock beneath sandy-silty overburden.

In seismic refraction work, only the first arrivals at each geophone were picked using Pickwin v 4.0 for shot gathers. First arrivals recorded from the multiple shots at each seismic array were employed to build the travel-time curves (Fig. 12a). The seismic measurements were processed and interpreted using SeisImager/2D software ^46^. In the SeisImager package, the applied inversion technique depends on an initial model in which seismic velocity (Vp) increases with depth, and in which the least-square scheme was then used. The initial velocity section was constructed after inverting the travel-time curves, whose layers were visually assigned (Fig. 12b). Finally, nonlinear travel time tomography was iteratively carried out to get the final model until the travel time data fit the perturbed initial model. In addition, the inverted velocities were presented using the ZondST2D program ^47^ to image the bedrock discontinuities along each profile. While this program was designed using smoothing Inversion applied to ensure model smoothness and focus to get a piece smoothed model of velocities with depth, The RMS error ranged from 2.5 to 3.4, which is considered acceptable and relatively low, indicating that the inversion produced a reliable velocity model.

## Data Integration

The integration of geophysical and geotechnical datasets was conducted through a structured workflow designed to ensure consistency and minimize interpretational ambiguity. The process began with DC resistivity soundings, which provided the initial 1D subsurface models. These were refined using genetic algorithm (GA) inversion, a technique that reduces non-uniqueness by exploring multiple starting models within a broad search space. The refined outputs were stitched into continuous resistivity sections and cross-validated with borehole lithology, ensuring that modeled boundaries corresponded with observed stratigraphy.

To capture lateral heterogeneity and vertical structures, 2D electrical resistivity tomography (ERT) profiles were employed. These profiles were particularly effective in delineating faults, cavities, and fractured zones that could not be resolved by 1D soundings alone. In parallel, seismic refraction data constrained bedrock depth and velocity contrasts, providing complementary information on subsurface stiffness and continuity. Borehole records served as calibration points throughout the workflow, linking resistivity and velocity anomalies directly to lithological units and strengthening the reliability of the integrated interpretation.

Conflicts between datasets were resolved through an iterative process. For example, when resistivity suggested conductive clay while seismic velocities indicated competent limestone, borehole evidence was prioritized and inversion parameters adjusted accordingly. Similarly, in cases affected by suppression or equivalence effects in resistivity, seismic velocity data and borehole logs were used to fix model parameters such as depth or resistivity values. This reconciliation reduced ambiguity and ensured that the final subsurface sections were geologically consistent.

**Fig. 13:**
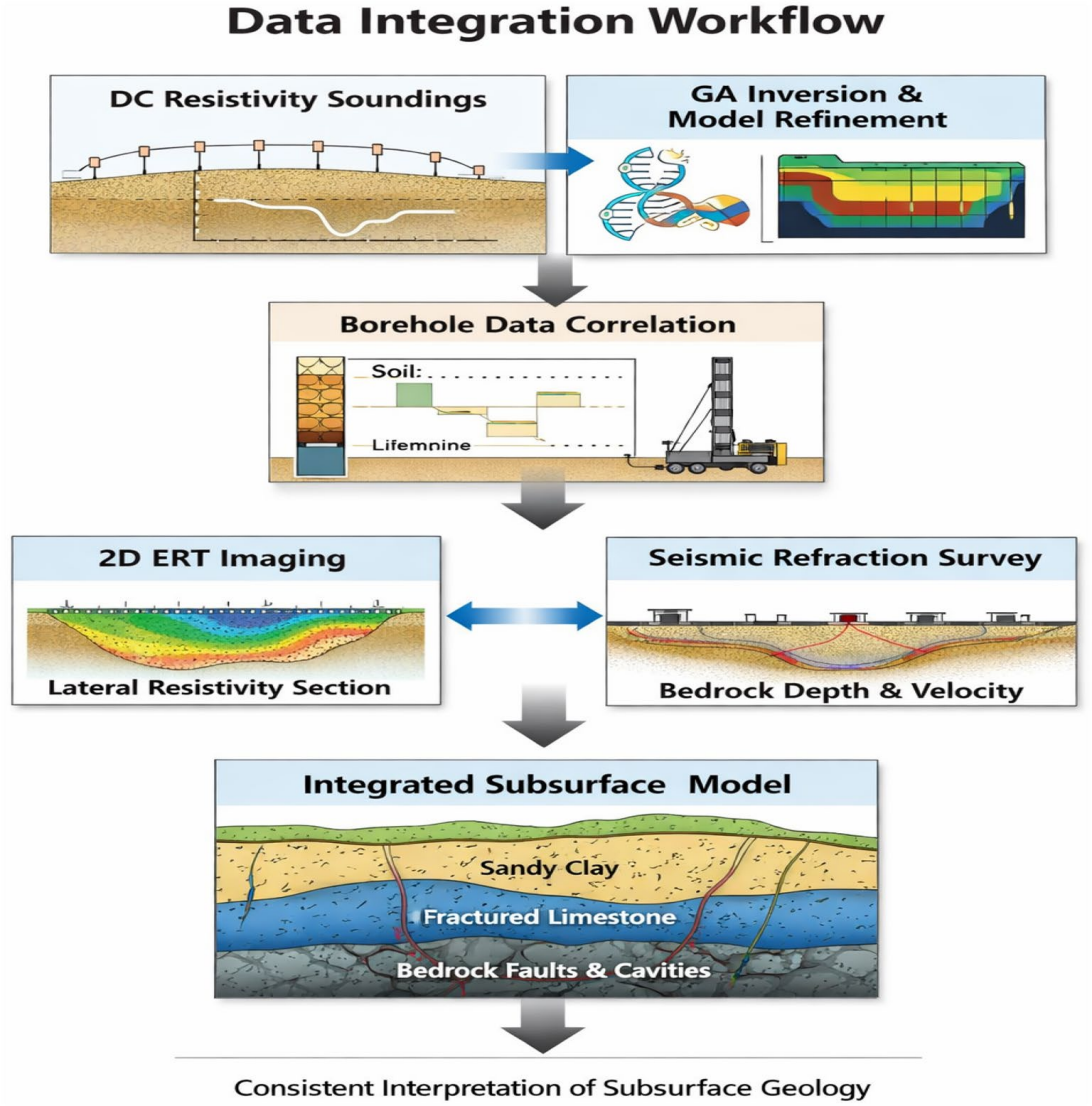
Integrated workflow diagram for New Minia site characterization, combining DC resistivity soundings, GA-based inversion, borehole calibration, 2D ERT imaging, and seismic refraction surveys to produce a unified subsurface model of New Minia City.

The integrated workflow produced robust subsurface models that delineated three principal sequences: sandy–silty overburden with clay intercalations, heterogeneous intermediate deposits, and fractured limestone bedrock. Major structural features, including north–south trending faults and karstic features, were clearly identified. By combining geophysical imaging with borehole calibration, the approach enhanced subsurface characterization, reduced uncertainty, and provided a reliable framework for geotechnical site investigation and hazard assessment in New Minia City (Fig. 13).

Direct borehole observations, including lithology descriptions, Rock Quality Designation (RQD), Uniaxial Compressive Strength (UCS), and Standard Penetration Test (SPT) values, are treated as the fundamental ground-truth constraints in this study. These parameters provide direct, site-specific evidence of subsurface conditions and serve as benchmarks against which geophysical interpretations are calibrated. In contrast, geophysical inversion results generate interpolated and extrapolated subsurface models that extend understanding beyond the discrete borehole points, offering continuous coverage across the investigated area. By maintaining this distinction, the manuscript ensures that measured data are not conflated with inferred information, thereby preserving methodological transparency and reinforcing the reliability of the integrated interpretation.

## Results and discussions

### Bedrock discontinuities from resistivity sounding

**Fig. 14:**
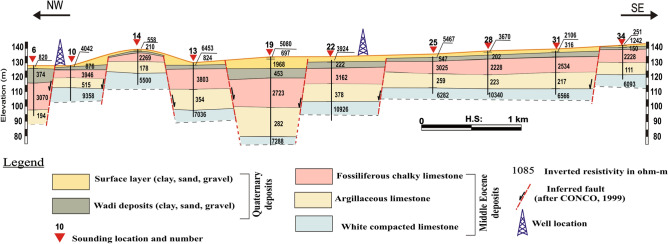
The constructed stitched resistivity sections resulted from DC resistivity soundings inversion controlled by outcrop and borehole database.

To illustrate the lateral variations and lithologic discontinuities, the resistivity–depth models obtained from the measured soundings were stacked together to obtain a quasi-2D section along five profiles in the area. Profile A–A is presented as an example in Fig. 14. This approach is expected to give reliable results where considerable variations occur in the limestone bedrock at foundation depth, and the profile line readings are assumed to represent a reflection of the geological section. The inverted resistivity section (Fig. 14) shows high-resistivity anomalies at shallow depths, with sharp boundaries between cavernous limestone bedrock and wadi deposits. Comparison between the resistivity models and borehole data indicates that discontinuities in the limestone bedrock are laterally controlled by faults and fractures, as well as by the presence of clay and fine deposits. Depths to limestone bedrock identified at boreholes and through resistivity models are relatively consistent across most borehole sites, supporting the reliability of the interpretation.

Based on the inversion results of the DC resistivity soundings, representative stitched resistivity sections (Fig. 14) were constructed to delineate the regional geological, structural, and hydrogeological settings, with particular attention to the fault-related dome structure at the study site. The integration of abundant borehole information helped to overcome ambiguities inherent in DC resistivity soundings and provided a reliable connection between geological observations and interpreted resistivity data. Special emphasis was placed on the lithology, thickness, and depth of the geoelectrical layers, given their critical role in detailed subsurface geological and structural mapping. From a structural perspective, abrupt changes in resistivity values and layer thicknesses (Fig. 14) are attributed to rock deformation processes. When compared with borehole records and outcrop geology, the stitched resistivity sections consistently support the construction of problem-solving cross-sections that illustrate the overall structural geometry of the study site, following the approach of ^48^.

It is important to emphasize that electrical resistivity and seismic velocity represent fundamentally different electrical and elastic physical properties and therefore direct comparison between them without appropriate constraints may lead to ambiguity. In this study, resistivity ranges were primarily derived from 2D-ERT results rather than relying solely on 1D inversion, thereby minimizing uncertainties associated with interpolation effects and lateral heterogeneity. To improve the reliability of lithological interpretation, the geophysical results were carefully calibrated against shallow borehole data, ensuring that resistivity values were consistently linked to corresponding lithological units. Based on this correlation, Table 1 has been revised to present a more coherent relationship between geophysical parameters (resistivity and velocity) and geotechnical properties. Nevertheless, the characterization remains conceptual due to the inherent differences between geophysical parameters and soil classification systems.

**Table 1:** Correlation between geophysical and geotechnical properties of the geological materials in the study area.

Geological classification	Resistivity (Ω.m)	Velocity, *Vp* (Km/sec)	RQD	UCS	SPT	W (%)	PI	Geotechnical description
			(%)	(Kg.cm^2^)	N value			
Limestone fragments with varying proportions of silty clay intercalation	1500–10,000	0.3–1.00	–	–	–	–	–	
Sand to clayey sand soil with limestone fragments	264–914	0.4–1.4	–	–	30-Oct	–	–	Medium dense
Silty clay with sand	40–150	–	–	–	–	14–29	8–19	Medium to High plasticity clay
Fractured fossiliferous limestone	717–3946	2.3–4.3	10–40	22–523	–	–	–	Very weak, weak and moderately strong rock

The inspection of 1D resistivity sections displays five geoelectric layers comprising the topsoil with resistivity varying from 251 to 61,412 Ω.m and thickness ranging from 0.5 to 1.5 m; the second is stream (wadi) deposits (sand and clay with limestone fragments) with resistivity varying from 50 to 1051 Ω.m and thickness ranging from 2 to 30 m); the third layer is fractured fossiliferous limestone (resistivity varies from 1000 to 5995 Ω.m and thickness ranges from 5 to 30 m); the fourth layer is argillaceous limestone (resistivity varies from 41 to 704 Ω.m and thickness ranges from 5 to 20 m) and fifth layer is white compact limestone (resistivity varies from 5034 to 11,428 Ω.m.

**Fig. 15:**
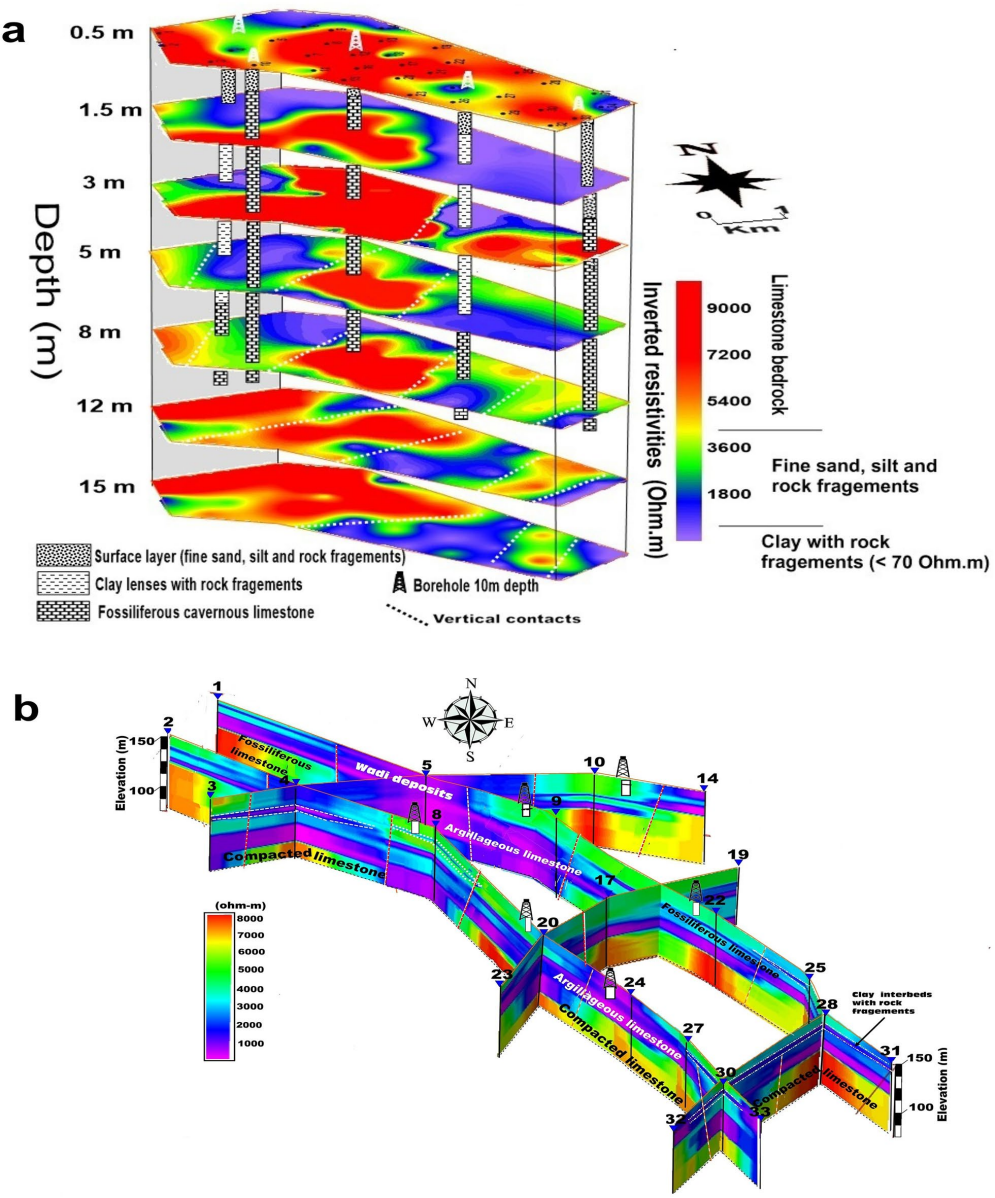
The vertical and horizontal resistivity distribution from resistivity sounding survey (**a**) Resistivity slicing maps at various depths. (**b**) 3D visualization fence diagram showing the distribution of resistivity with depth along some definite directions in the Study area.

To visualize the horizontal and vertical variations in the subsurface in a 3D view, the 1D inverted resistivities were used to generate slicing maps at different depths (Fig. 13a) and a 3D fence model of the entire area (Fig. 15b). The output 3D visualization model was created by employing Rockworks’ inverse distance–anisotropic algorithm 14 package ^41^.

According to the layer distribution in the study area, seven resistivity maps were constructed from the ground surface to 15 depths to illustrate the lateral and vertical variation of resistivity with depth (Fig. 15a). The correlation of the interpreted resistivity slicing maps indicates that there are a general lateral and vertical heterogeneity all over the study area, reflecting soil and bedrock discontinuities. The surface layer represents high resistivity values (123 – more than 10,000 Ω.m). The resistivity range of this layer may be explained by the clay content. The local low resistivity anomalies (blue color) around some soundings (e.g., 18, 24, and 34) can be attributed to clay and clayey sand bodies. The relatively low resistivity value (77 to 200 Ω.m) corresponding to the silty clay layer to argillaceous limestone (the blue color) is featured at 12 m depth side by side with a high resistive layer (fractured fossiliferous limestone), which reflects the lateral heterogeneity. Fractured limestone layer (red color, 1000 to 4000 Ω.m) appears at various depths and extends vertically to the last slice. It appears shallower (3 m depth) at the western part and NW direction, and extends vertically to the last slice and laterally to the NE direction at 15 m depth. The 3D fence model (Fig. 15b) reveals that there is a lateral and vertical heterogeneity, which is mainly structurally controlled as indicated by the linear boundaries between high and low resistivity anomalies.

### Imaging of rock discontinuities.

**Fig. 16:**
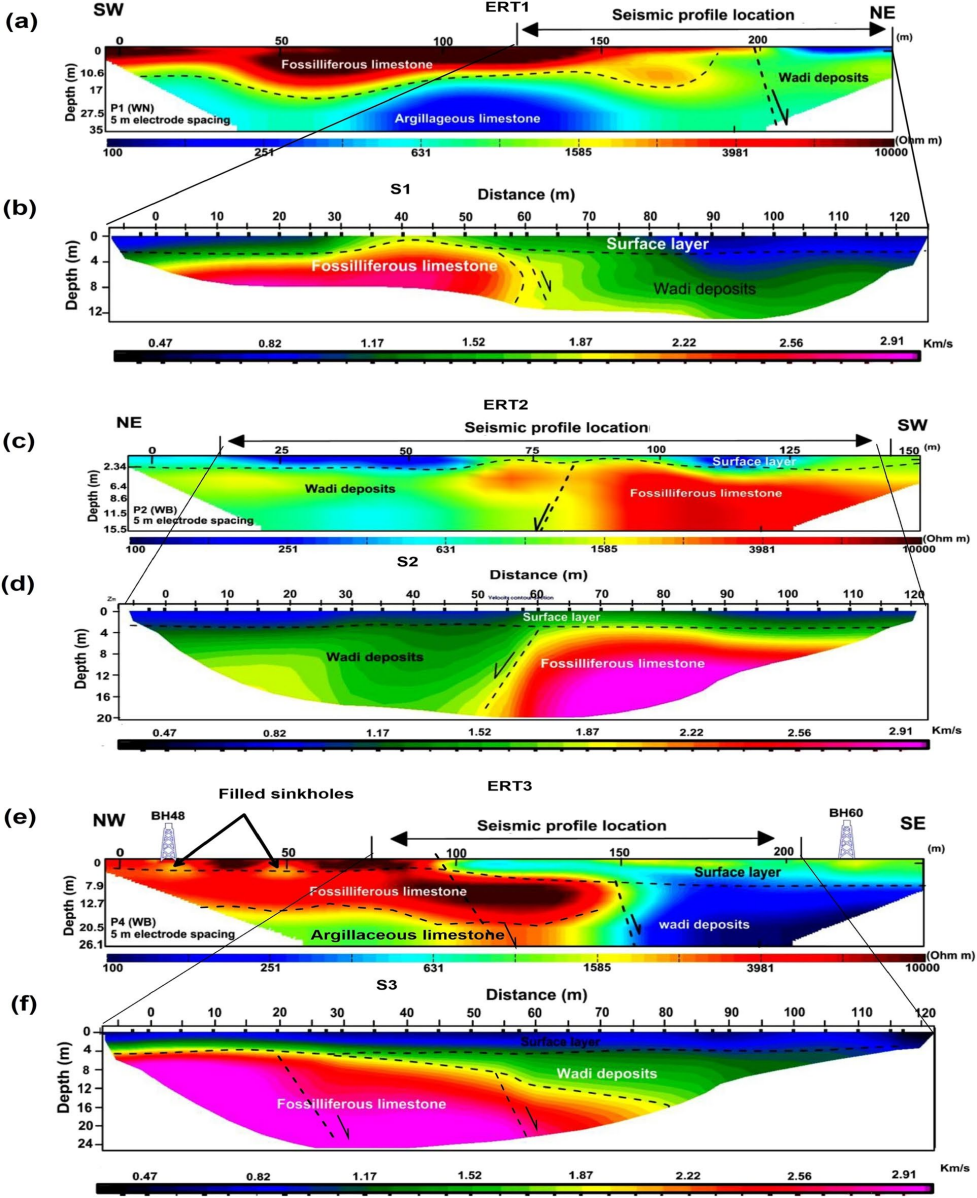
2D inverted ERT and seismic refraction sections of profiles 1, 2 and 3 show the bedrock discontinuities in the area (for location see Fig. 7).

**Fig. 17:**
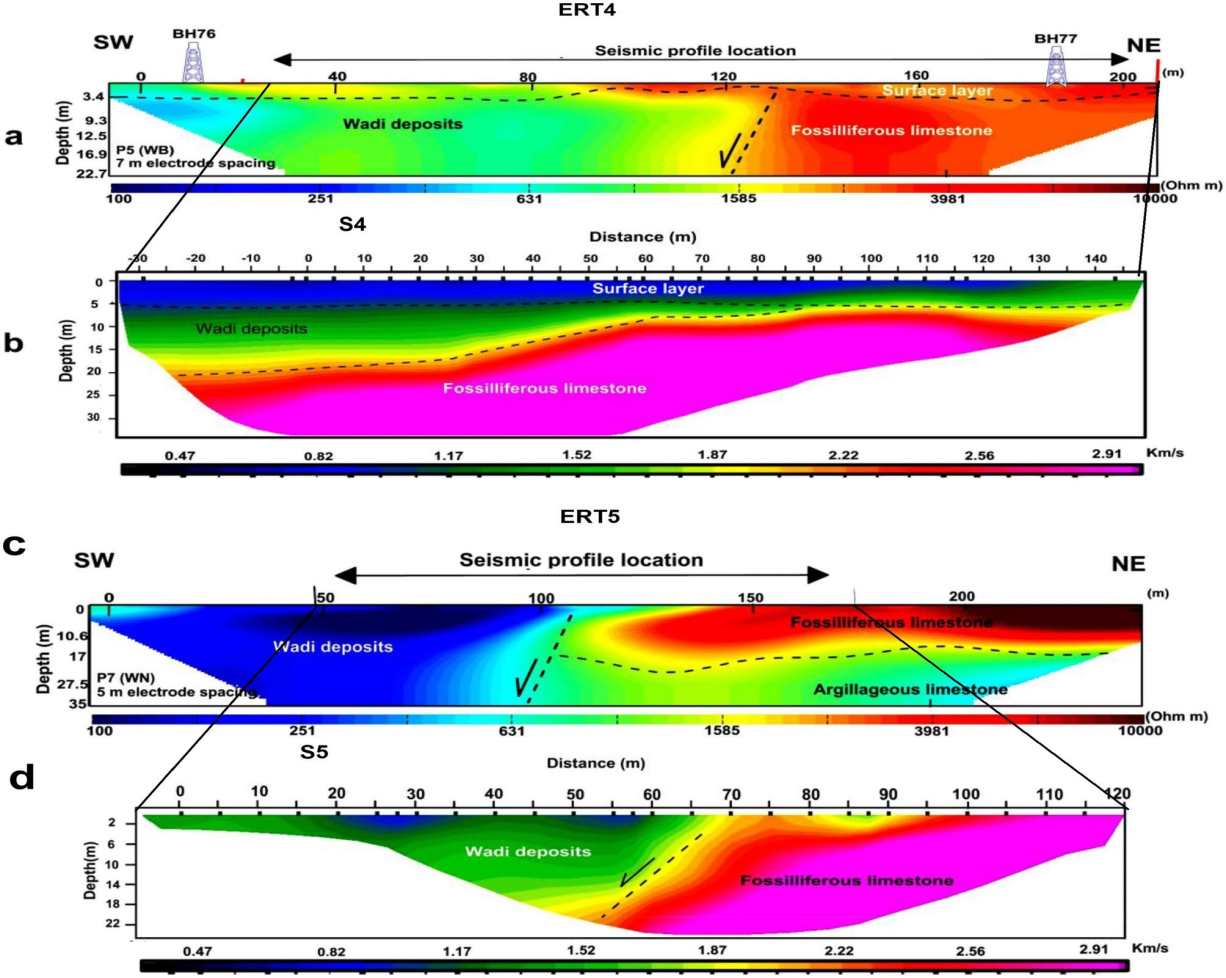
2D inverted ERT and seismic refraction sections of profiles 1, 2 and 3 show the bedrock discontinuities in the area (for location see Fig. 7).

Electrical resistivity tomography (ERT) and seismic refraction imaging can aid in interpreting failure zones, lithologic differences, and changes in moisture conditions in the foundation bedrock. The 2D resistivity tomograms were used to map the short-distance discontinuities and depth to bedrock, where the field observations and borehole information were employed to support the interpretations of the inverted images. The joint application of these procedures is necessary in order to improve the accuracy of the survey, where each method presents different sensitivity and limitations, according to the physical properties of the common materials in the surveyed area. Therefore, the inspection of the obtained 2D ERT and seismic refraction images displays a close match for mapping rock discontinuities in the limestone bedrock (Figs. 16 and 17).

ERT was selected due to the depth of investigation required (up to 30 m) and the presence of conductive clay interlayers. Seismic refraction complements ERT by resolving velocity contrasts, but it is less sensitive to thin low-velocity layers. The combined use of ERT and seismic refraction thus provides a balanced approach to mapping discontinuities in fractured limestone.

As shown in Fig. 14a, the inverted 2D resistivity profile (ERT1) shows a high resistivity layer (> 1500 Ω.m) from the beginning of the profile to 190 m in length, corresponding to fossiliferous dense limestone bedrock. This high resistivity layer extends to ~ 17 m depth. In contrast low to moderate resistivity layer (100–600 Ω.m) that represents the argillaceous limestone was observed underlying the fractured fossiliferous limestone. At a horizontal distance of 160 m from the start point, the high resistivity layer (fractured fossiliferous limestone) changes laterally into a medium resistivity layer corresponding to fine alluvial deposits. These lateral bedrock discontinuities can be attributed to faulting. By considering the length and vertical scale, the visual inspection of the velocity-depth model, S1 (Fig. 16b) shows quite uniform velocity with an average velocity of 0.692 km/sec and a thickness of ~ 2 m for the first layer (surface layer). The second layer represents an average elastic wave velocity of 2.231 km/sec corresponding to fractured fossiliferous limestone, which changes laterally into a medium velocity layer (wadi deposits) with an average velocity of 1.5 km/sec. Both electrical and velocity Images (Fig. 16a and b) show a lateral discontinuity along the measured profile, which in turn reflects that great care should be taken in this part of the site prior to building works. In the same manner, the abrupt change in resistivity values along the 2D profile, ERT2 (Fig. 16c), can be attributed to rock discontinuities and/or fault existence. The location of this vertical contact (at 85 m distance) was confirmed through seismic profile S2 (Fig. 14d). The middle part at a distance of 60 m shows a good match with the ERT2 profile. Additionally, in the inverted sections of ERT3 and S3 (Fig. 16e and f, respectively), outstanding resistive vertical bedrock was clearly identified in the western side of the profile at shallow depth with a sharp vertical discontinuity at 150 m distance, which could be matched with seismic results. Towards the eastern side of the vertical contact (Fig. 16e) The dashed black lines seem to coincide with resistivity discontinuities between the high conductive zone and the lower to medium resistivity layer (surficial dry soil and the fine materials of the alluvial deposits, respectively). Zones of anomalously low resistivity, interpreted as clay-filled caves, are explicitly marked in Fig. 14e, highlighting structurally weakened intervals that require careful engineering consideration. In the velocity section (Fig. 16f), the mean velocity of the stream deposits was 1.2 km/sec, which changes laterally to a high velocity layer (2.9 km/sec) reflecting the fractured fossiliferous limestone bedrock. So, there is a great variation in both resistivity and velocity due to the NW–SE faulting trend. Another type of bedrock discontinuity can be noticed in the western side of the profile as low resistivity sinkholes in high resistivity background (at 25 and 50 m distance and 2 m depth, Fig. 16e). Therefore, these near-surface sinkholes and caves which are filled with fine and clay materials, may increase the hazardous situation in the area and change the stability of the limestone bedrock due to the relative decrease of shearing resistance.

The presented ERT results are promising and align with anomalies reported in karstic terrains elsewhere ^20,49^. In particular, comparable low-resistivity signatures have been documented over known caves and sinkholes, confirming the reliability of ERT in detecting subsurface voids. However, caution is warranted, as similar anomalies may also represent saturated clay pockets or water-filled cavities rather than true karst features ^50^. By comparing both the resistivity ranges and anomaly geometries with published case studies, the present interpretation acknowledges similarities while also highlighting potential sources of misinterpretation. This comparative approach strengthens the reliability of the geophysical analysis while avoiding overstatement of results.

Additionally, the inverted sections of ERT and the velocity model of profiles 4 and 5 (Fig. 17) display a good correlation and give enough evidence on the bedrock discontinuities, and in addition, which is visible as a sharp contact between resistive bedrock and relatively low resistivity clay, sand, and fine deposits. However, ERT demonstrates superiority over the seismic refraction method to detect the low velocity layer noticeable at greater depth where the resistivity decreases with increase in depth at 15 m of ERT3 and ERT5 (Figs. 16e and 17c, respectively), which shows obvious low to moderate resistivity due to the argillaceous limestone layer. This layer has totally disappeared in the seismic section and is suppressed by the upper bedrock (2.9 Km/sec) due to the hidden layer problem, where the overlying dense fossiliferous limestone has a higher velocity than the lower argillaceous limestone bedrock. Subsequently, the combined use of seismic refraction method and ERT approach is employed as a comprehensive imaging tool to detect the hidden subsurface structures that represent potential risk to infrastructure and lives in the area.

The integration of DC resistivity, seismic refraction, and borehole calibration in New Minia City provides a comprehensive framework for subsurface characterization in fractured limestone terrains. Similar integrated approaches have been reported in carbonate settings worldwide (e.g., Martínez-Pagán et al., 2020; Abidin et al., 2023), where combining electrical and seismic methods reduced interpretational ambiguity and improved hazard assessment. Our study contributes to this body of work by applying genetic algorithm (GA) optimization to resistivity inversion, which enhanced model convergence and reduced non-uniqueness compared to conventional linear filter inversion.

Despite these advances, several limitations must be acknowledged. First, the maximum depth of borehole control (10 m) restricts calibration of deeper anomalies, meaning that interpretations below this depth rely primarily on geophysical inversion. Second, seismic refraction is less sensitive to thin low-velocity layers between high-velocity strata, which may obscure subtle discontinuities. Third, resistivity inversion is subject to equivalence and suppression effects, particularly in clay-limestone sequences, which can complicate layer resolution. Finally, GA optimization, while reducing non-uniqueness, is computationally intensive and requires careful parameterization to avoid overfitting.

Each methodology demonstrated distinct strengths and limitations in terms of accuracy. DC resistivity soundings with GA inversion achieved strong correlation with borehole lithology, with RMS errors as low as 3.46 (VES24), indicating reliable model fitting. ERT profiles provided high-resolution imaging of lateral heterogeneity and vertical structures, successfully delineating faults and cavities that were not visible in 1D soundings. Seismic refraction accurately constrained bedrock depth and velocity contrasts, complementing resistivity data by providing stiffness information, though its resolution decreased in highly heterogeneous zones. Borehole calibration anchored geophysical anomalies to physical lithologies, ensuring geological consistency, but was limited by shallow depth and sparse distribution.

Overall, the integrated workflow improved accuracy by cross-validating datasets and resolving conflicts iteratively. The combined use of GA-optimized resistivity inversion, ERT imaging, and seismic refraction, supported by borehole calibration, provided a robust subsurface model that reduces uncertainty in foundation assessment.

### Geotechnical characterization of bedrock and soil

The inferred results of both resistivity and seismic refraction data were correlated with field observations and borehole information to fully understand the nature and risk of geologic discontinuities at foundation depth. Accordingly, the foundation bedrock in the area is composed of two layers besides the surface layer. The surface layer, which covers the area consists of a mixture of sand with limestone fragments, and sandy clay in some sites. The second layer is composed of sand, clayey sand, and sand containing limestone fragments that vary laterally into fossiliferous cavernous limestone bedrock. The correlation between geophysical and geotechnical data is listed in Table 1. According to this correlation, the soil (sandy clay) material is characterized by low resistivities of less than 150 Ω.m. The observed low resistivity is attributed to the elevated clay fraction and significant water content, which reached approximately 29% of the material. The moisture content affects the consolidation condition, Soil shear strength, and its suitability for compaction ^51^ and ^52^

Particularly, the consistency of fine-grained soil depends largely on its moisture content. The SPT (N) value of soils in the study area ranges from 10 to 30 at depths from 1 to 10 m, with a resistivity value reaching 900 Ω.m (Fig. 15). The increase in velocity from 0.7 km/sec to 1.3 km/sec with depth within the soil material can be explained by its clay content. In the surveyed area, the shallow fractured limestone bedrock was clearly defined with sharp vertical boundaries. It was characterized by resistivities of 1070 to 3946 Ωm and velocities of 2.034 to 4.357 km/sec.

The obtained results from the DC resistivity were integrated with the geotechnical tests, such as Unconfined Compressive Strength (UCS) and Rock Quality Designation (RQD) of bedrock. The UCS value of rock in the area ranges from 22 to 523 kg/cm^2^. Based on the standard description of UCS, rocks of the study area are grouped under very weak, weak, moderately, and strong rock. The obtained RQD values display the degree of fracturing in the limestone bedrock. The limestone bedrock has RQD values ranging from 10 to 25% to indicate very poor to poor foundation material according to the Egyptian code of foundation ^53^.

In contrast, the subsurface clay and fine deposits were clearly defined with resistivities less than 150 Ω.m. The decrease in resistivity and increase in velocity in some places are due to the clay content. The free swelling tests reveal that the sandy clay and clay soils are moderately swelling and probably cause engineering problems during construction or after the process of construction. In addition, the values of plasticity index (PI) range from 8 to 19, which correspond to medium to high plasticity should be prevented, where soils with a high PI tend to be clay, while those with a PI near zero contain very small amounts or no silt and clay ^54^. Most of these materials are concentrated at shallow depths (1–4 m) in the northern and western parts which are characterized by low resistivity anomalies (Fig. 15).

## Conclusion

The study demonstrates that integrating DC resistivity soundings, 2D-ERT, and seismic refraction data, calibrated with borehole information, provides a reliable framework for mapping subsurface discontinuities in fractured limestone. The combined approach successfully delineated lithological variability and structural features, including NW–SE trending faults and possible sinkhole zones.

Evidence from resistivity and seismic velocity contrasts shows that the central and western sectors are underlain by relatively competent limestone, while the eastern and northern zones contain fine-grained soils with higher plasticity, posing geotechnical risks. The application of GA-based inversion improved resistivity model reliability by reducing non-uniqueness and enhancing correlation with borehole data.

Overall, the integrated methodology reduces interpretational uncertainty and provides a robust basis for foundation design, hazard assessment, and urban planning in rapidly developing areas such as New Minia City.

## Data Availability

The datasets used and/or analyzed during the current study are available from the corresponding author on reasonable request.
